# Natural Variation in Decision-Making Behavior in *Drosophila melanogaster*

**DOI:** 10.1371/journal.pone.0016436

**Published:** 2011-01-20

**Authors:** Paige M. Miller, Julia B. Saltz, Veronica A. Cochrane, Caitlin M. Marcinkowski, Raisa Mobin, Thomas L. Turner

**Affiliations:** 1 Ecology, Evolution, and Marine Biology Department, University of California Santa Barbara, Santa Barbara, California, United States of America; 2 Center for Population Biology, University of California Davis, Davis, California, United States of America; 3 Molecular and Computational Biology Department, University of Southern California, Los Angeles, California, United States of America; University of California Davis, United States of America

## Abstract

There has been considerable recent interest in using *Drosophila melanogaster* to investigate the molecular basis of decision-making behavior. Deciding where to place eggs is likely one of the most important decisions for a female fly, as eggs are vulnerable and larvae have limited motility. Here, we show that many natural genotypes of *D. melanogaster* prefer to lay eggs near nutritious substrate, rather than in nutritious substrate. These preferences are highly polymorphic in both degree and direction, with considerable heritability (0.488) and evolvability.

Relative preferences are modulated by the distance between options and the overall concentration of ethanol, suggesting *Drosophila* integrate many environmental factors when making oviposition decisions. As oviposition-related decisions can be efficiently assessed by simply counting eggs, oviposition behavior is an excellent model for understanding information processing in insects. Associating natural genetic polymorphisms with decision-making variation will shed light on the molecular basis of host choice behavior, the evolutionary maintenance of genetic variation, and the mechanistic nature of preference variation in general.

## Introduction

*Drosophila melanogaster* is often considered the consummate generalist, as many different types of rotting fruit or vegetables can be used as rearing substrate [1], [2], [3]. Adults are highly motile [4], and can experience a wide variety of resources in their lifetime (e.g. a fallen fruit in an orchard, a compost pile in a suburban area, or a barrel of fermenting wine in a wine cellar [5]). In addition to the wide differences between available food patches, each patch is likely to be quite heterogeneous. Within a single rotting fruit, for example, the stochastic nature of colonization by bacteria and fungi may lead to considerable variation.0 Within this tremendously varied environment, a female fly must assess possible oviposition sites so that she can decide where to invest her most valuable resource: her eggs. Females demonstrate plasticity in their oviposition behavior and make choices that may benefit their offspring [6]. It was recently shown that flies undergo a search-like behavior even on homogenous lab medium, and probe possible oviposition sites with multiple sensory structures [7]. They also withhold eggs in the absence of quality oviposition media, further supporting the idea that females are choosy regarding their placement [3].

Yang and colleagues [7] recently proposed that oviposition-site selection is an excellent model for investigating the molecular basis of decision-making behavior. Surprisingly, these authors found that females from the Canton-S lab line preferred to oviposit on sucrose-free media when presented with media containing 1% agar, 1% ethanol, +/- 1% sucrose. Though *Drosophila* females clearly need to deposit their eggs in a location that will promote larval development, environments such as rotting fruit may contain patches of nutritious substrate interspersed with areas that offer developing embryos refuge from microbial decomposition. For example, on fruits that have just started to decompose, we have observed that *D. melanogaster* females will deposit eggs into the stem cavity in addition to directly ovipositing into a rotting abscess. If genetic and environmental variation affects these decisions (as supported by previous studies of oviposition behavior in *Drosophila* [6], [8], [9], [10], [11], [12], [13], [14], [15], [16], [17], [18], [19], [20], [21], [22]), this variation could be used as a model for individual differences in preference behavior. Here we show that natural isolates of *Drosophila melanogaster* have surprising preferences when presented with simple media containing acetic acid, ethanol and agar, with or without yeast extract. Though yeast extract contains important nutrients for developing larvae [23], females of many genotypes prefer to oviposit in media lacking yeast extract, but only when the site is in close proximity to nutritious media. This behavior requires the integration of multiple information sources, and therefore presents an interesting opportunity to investigate the mechanistic basis of decision-making. Moreover, this behavior varies greatly between genotypes, and is affected by environmental variables such as the overall concentration of ethanol. By adapting an apparatus from Joseph et al. [24], we have quantified these decisions in thousands of individuals, and established a system that can be used for genome-wide association studies of decision-making behavior.

## Results

To investigate genetic variation in oviposition-related decisions, we used a two choice assay: two media were poured side-by-side in a 35 mm petri plate lid, and this lid was presented overnight to single mated females inside a 170 cc plastic arena. One option consisted of 1% agar with 0.8% ethanol and 0.8% acetic acid: ethanol and acetic acid are byproducts of microbial metabolism which are attractive to *D. melanogaster* at these doses (see below), but this substrate contains little nutritional value. The second option presented was the same, with the addition of highly nutritious yeast extract (1%). These options are intentionally simple: preliminary experiments suggested that this 4-ingredient media (agar, ethanol, acetic acid, +/- yeast) is one of the simplest substrates females will accept. We expect that this assay (2 options within a single 35 mm plate) is similar to the decision-making process faced by females when confronted with a single heterogeneous patch, such as a rotting fruit. This experiment was designed not to mimic the natural environment precisely: we attempted to create a simple, efficient assay that can assess behavioral variation in a large number of genotypes and environments. As such, behaviors in this assay could serve as a simple model system to dissect natural variation in the insect decision-making circuit.

### Genetic variation in decision-making behavior

**Figure 1:**
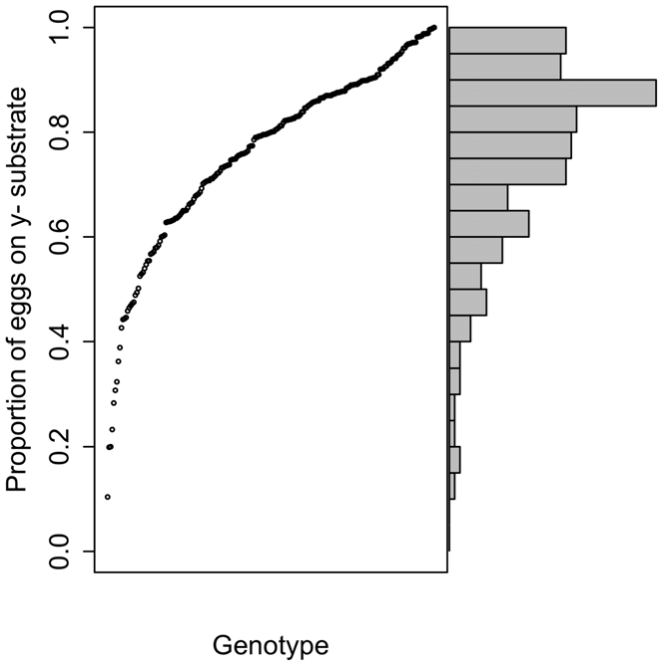
The average proportion of eggs laid on y^-^ media for 213 inbred lines. Variance and sample sizes vary considerably between genotypes (see text), and only means are shown for each genotype, for clarity. On the right, a histogram of the same data is shown. Minimum sample size per line  = 5 females, mean  = 11.6 females.

To assess the extent of genetic variation in oviposition decision-making, we assayed a total 5187 flies from 295 natural (“wild-type”), inbred genotypes. As some genotypes were reticent to oviposit on either option, five or more replicate females were assessed from only 213 of these genotypes (mean replicates  = 11.6). The average preference for each of these 213 genotypes is shown in figure 1. Ninety-six of these 213 genotypes were collected at a fruit market in Raleigh, North Carolina (by T. F. C. Mackay [25]), and 117 were collected from a fruit orchard in Winters, California (by S. Nuzhdin [26]): the trait values of the two populations are not significantly different (Raleigh mean  = 0.775, Winters mean  = 0.750, *t* = 1.04, *p* = 0.30), so all genotypes were considered together.

The average proportion of eggs laid on yeast-free (y^-^) medium varied considerably between inbred lines, with one line laying 100% of eggs on y^-^ media in each of 11 replicates, while at the other extreme a line averaged only 10.4% of eggs laid on this option. Most lines had less extreme preferences, laying some eggs on each substrate, but preferring y^-^: only 9% of genotypes averaged <50% on y^-^. To estimate the proportion of variation in this trait that results from genetic variation, we partitioned the variance into within- and among-line components using sum of squares. The among-genotypes component of variance, which provides an estimate of the broad-sense heritability, is found to be considerable: 48.8%. As variance is not homogenous across lines (some lines have near zero variance), the proportional data are very non-normal, and sample sizes differ across lines, we determined the statistical significance of the among-genotype variance by resampling data 100,000 times and partitioning variance among this permuted data. Permuted data sets averaged 9.4% among-genotype variance, with no values greater than 14%; the observed value of 48.8% is therefore clear evidence of substantial genotypic variation in decision-making (*p*<1.0e10^−5^).

**Figure 2:**
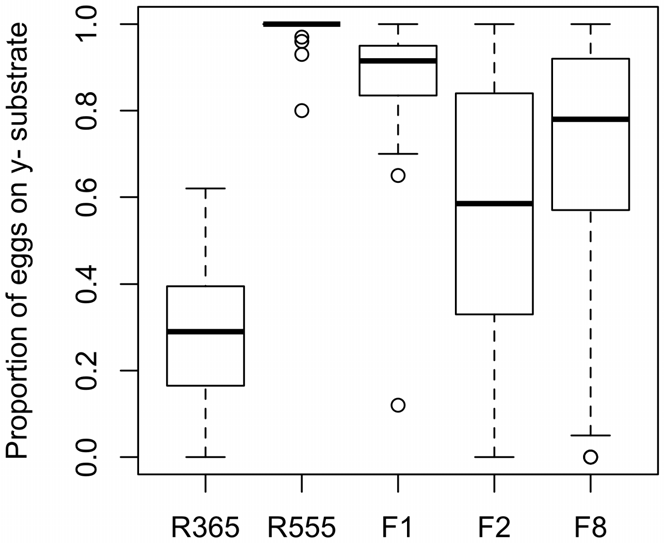
The distribution of preferences among females of two RAL genotypes (R555 and R365), their F1 offspring (with R555 as the maternal parent), and their F2 offspring. Median (line), 75% quartile (box), and range excluding outliers (whiskers), and outliers (circles) are shown.

The among-genotypes component of variance estimates the total contribution of genetic variation, including additive, dominance, and epistatic components. To characterize this variation further, we crossed two of the most extreme genotypes from the Raleigh (RAL) collection of lines. The RAL-555 line averaged 99% of eggs on y^-^: of the 486 eggs laid over 31 replicates, this line laid only 4 eggs on the y^+^ option. In contrast, RAL-365 laid 270/397 eggs on y^+^ media, averaging 28.4% of eggs on y^-^ across 20 replicates (fig. 2). Trait values of F1 genotypes reveal that preference for y^-^ is largely dominant, with 97% of F1 individuals preferring y^-^ more than the midparent value. Preferences do not show complete dominance, however, as only 12.5% of F1 individuals laid 100% of eggs on y^-^, and the distribution of F1 and RAL-555 preferences are significantly different (Wilcoxon *p*<1.0e^−7^). In contrast, the distribution of 346 F2 individuals averaged 57% of eggs on y^-^, near the mid-parent value of 63.5%. This may indicate that the apparent dominance seen in F1 genotypes is due to epistasis, or that there are threshold effects when phenotypes are near the boundary of 100%. In any case, these data are consistent with quantitative variation in oviposition decision-making.

**Figure 3:**
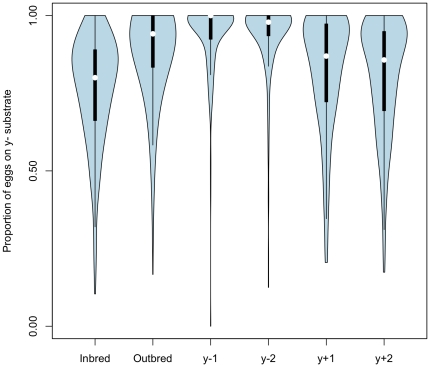
Violin plots of preference behavior. Each element contains a Tukey box plot showing the median (white dot), 75% quartile (thick line) and range excluding outliers (thin line). Surrounding the box plot is a kernel density trace, plotted symmetrically on both sides of the boxplot, which provides a graphical comparison of each distribution (following [34]). Inbred  =  distribution of inbred RAL genotypes; Outbred  =  the F_8_ population started from these inbred lines; y-1 and y-2 =  populations selected for a single generation in the direction of 1.00; y+1 and y+2 =  populations after two generations of selection in the direction of 0.00.

We used artificial selection to directly assess the evolvability of oviposition preferences. An outbred population was created by cross-mating virgin females and males from 173 inbred lines from the RAL collection (see methods). After eight generations of stochastic recombination, we quantified the preferences of 863 outbred individuals from this population. Consistent with partial dominance, the average preference of these outbred F_8_ individuals was biased more towards the y^-^ substrate compared to the distribution of inbred lines (inbred mean  = 0.76, outbred mean  = 0.89; fig. 3). We then divided these 863 females into four populations: those with preferences above the median value were divided into two populations to start selection in the y^-^ direction, and those below the median were used to found two populations in the y^+^ direction. As shown in figure 3, selection in the y^-^ direction was immediately and dramatically successful. When realized heritability is calculated as the ratio of the response to selection over selection differential, heritability in this direction is estimated to be slightly more than 1.00 (realized h^2^ = 1.10,1.02 for y-1 and y-2 populations, respectively). In the y^+^ direction, we selected for two generations: average realized heritability over these two generations was 0.66 and 0.81 for each population.

### Egg number

For the quantification of preference behavior discussed above, we discarded data from females who laid fewer than 5 eggs, as preferences were difficult to assess in these individuals. In preliminary trials with a small number of genotypes, these individuals represented a small proportion of the females tested. As more genotypes were tested, however, we found that the number of eggs laid was highly variable among genotypes,and that females of some lines never laid eggs in this assay.This variation could be due to many factors, but may in part reflect differences in acceptance of oviposition media betweenlines.

**Figure 4:**
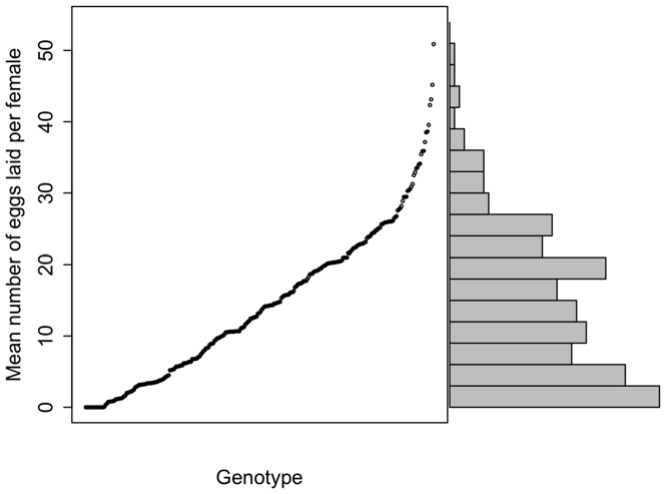
The average number of eggs laid for 282 inbred lines. Minimum sample size per line  = 5 females, mean  = 16.1 females. Variance and sample sizes vary considerably between genotypes (see text), and only means are shown for each genotype, for clarity. On the right, a histogram of the same data is shown.

**Figure 5:**
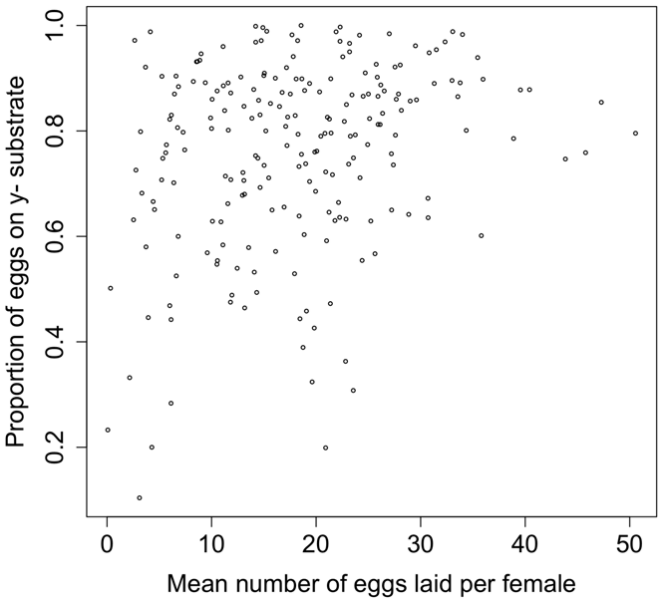
The relationship between average egg number and average preference for each line.

Of the 282 genotypes for which 5 or more females were presented with the assay, females from 15 genotypes never laid a single egg in any replicate. As these lines all oviposit on standard *Drosophila* culture media, this may indicate that both of the substrate options presented are considered inadequate by these genotypes: rather than choosing between y^-^ and y^+^ media, these females may be withholding eggs from both options in deference to potential future options. Figure 4 shows the distribution of average egg number among genotypes: when partitioning with sum of squares, 37.3% of this variation is among genotypes, indicating significant genetic variation for egg number (maximum of 100,000 permutations  = 7.40%, *p*<1.0e10^−5^). Egg number was significantly correlated with preference among these lines (r = 0.26, *p* = 1.1e^−4^), suggesting that some genetic polymorphisms affect both traits (fig. 5). The modest value of the correlation, however, suggests that these traits are also largely independent.

### Distance Effects

To explain the preference of most genotypes for non-nutritious media, we hypothesized that females of these lines choose to oviposit near yeast, rather than choose to avoid yeast altogether. Under this hypothesis, preference for y^-^ media in these lines would be eliminated if media containing yeast were not adjacent. We therefore designed an additional experiment, wherein the preferences of four genotypes were tested with increased distances between the y^+^ and y^-^ media. For this assay, the media was prepared in the same manner as above, but the two substrates were then moved to a large (150 ml) square petri plate. The two substrates were either placed in contact with one another in the center of the plate (0-cm distance), or a gap was left between options (1-cm to 8-cm distance). Mated females from each genotype were then allowed to oviposit in each petri plate overnight. We assayed four RAL genotypes with a range of preferences from the bottle assay (y^-^ proportions 0.989, 0.774, 0.475, and 0.284). Four females from each genotype were used in each replicate, rather then a single female, to increase the proportion of replicates with large numbers of eggs.

**Figure 6:**
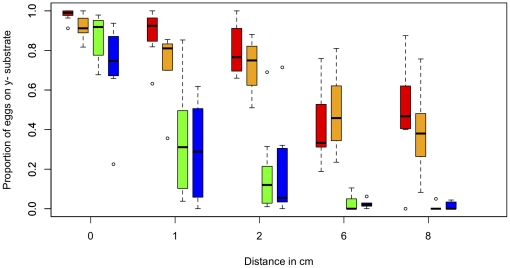
The effect of distance on oviposition preference. A Tukey box plot is shown for each genotype * distance combination; red  =  RAL-555, orange  =  RAL-437, green  =  RAL-208, blue  =  RAL-365.

In all genotypes, the distance between y^-^ and y^+^ media had a considerable effect on oviposition behavior. When analyzing each genotype independently, distance explained 63%–78% of the variance in each line (fig. 6; permutation performed as explained above, *p*<1.0e^−5^ in each case). For the two lines that oviposited primarily on y^-^ in the bottle assay (red and orange in fig. 6), the proportion of eggs on the y^-^ media decreased with distance until, at 8-cm, females laid approximately half of all eggs on y^+^. For two lines that did not prefer y^-^ in the bottle assay, distance had an even more dramatic effect, with nearly all eggs on the y^+^ media by 6-cm.

To determine whether these genotypes respond significantly differently to distance, we fit a Generalized Linear Mixed Model (GLMM) using SAS Proc GLIMMIX (v9.2; SAS Institute, Cary, NC 2009). Because the data were proportions, a binomial distribution and logit link function were specified. Initial tests indicated that genotypes differed significantly in variance (test of homogeneity based on residual pseudo-likelihoods: χ^2^ = 168.55, p<0.0001), so the model specified individual covariance parameters for each genotype [27]. The resulting model showed no evidence of overdispersion (Generalized χ^2^/DF  = 1.00). As genotypes were chosen non-randomly after the initial screen of 213 genotypes, genotype was considered a fixed factor in the analysis. The distance between oviposition choices was a continuous fixed factor. Denominator degrees of freedom for F-tests were estimated using the Kenward-Rogers method, which is appropriate for models with complex covariance structures [28], [29].

The GLMM verified a strong effect of distance on the proportion of eggs laid on the y^-^ side (F_1,83.5_ = 29.68, p<0.0001). Specifically, odds ratio estimates showed that females were 25.5 times (95% CL: 14.6–44.2) more likely to lay on the y^-^ side when the two oviposition substrates were adjacent than when they were 8 cm apart (fig. 6). The GLMM also confirmed the strong effect of female genotype on oviposition behavior (F_3,83.41_ = 5.41, p = 0.0019). Further, a significant genotype-by-distance effect illustrated that genotypes differed in how the distance between choices affected their oviposition preferences (F_3,77.97_ =  3.65, p = 0.018).

Surprisingly, at the 0-cm distance, the range of preference variation across genotypes was compressed in the petri-plates compared to the bottle assay. While average preference of the five genotypes tested varied from 28%–99% of eggs on y^-^ media in the bottle assay, the same lines varied from only 73%–98% in the petri plates. This effect was not due to changing the number of females in the assay from one to four: we assayed these genotypes with four flies in the bottle assay, and found no significant differences compared to the single-female data (permutation *p*>0.05 for all genotypes). Instead, this difference seems to be due to an unknown effect of assay condition, as two of the four lines tested have significantly different preferences between bottle and plate assays (Bonferonni-adjusted permutation *p*<0.05). The 150 ml petri plate is of a similar volume as the bottle assay (177 ml), so the shape of the container would seem the primary difference between them. Despite this assay-effect, preferences were highly correlated between assays (r = 0.94), though with only four lines tested, this correlation is only marginally significant (*p* = 0.06).

### Other environmental effects

The modification of oviposition-related decisions with distance suggests that flies integrate multiple sensory modalities when choosing where to oviposit. To further investigate the plasticity of these decisions, a subset of genotypes was tested at different concentrations of ethanol and acetic acid (while keeping the other ingredients in the media, water and agar, at standard concentrations).

**Figure 7:**
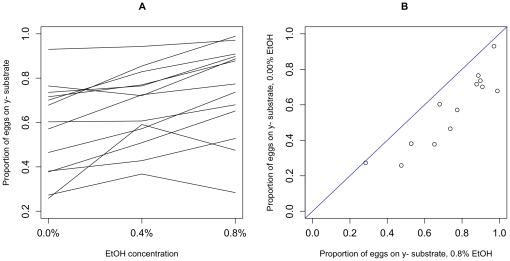
The effect of ethanol on oviposition preference. A: The effect of ethanol on behavior towards yeast is shown for 13 RAL genotypes at three concentrations of ethanol, where each line is a genotype. B: Plot of mean preference of genotypes in A, at two ethanol concentrations, shows that preferences are highly correlated between ethanol concentrations, with more avoidance of yeast substrate at higher ethanol concentration.

When the amount of ethanol in both oviposition substrates is simultaneously reduced, all genotypes oviposit more eggs on the y^+^ media (fig. 7). To quantify this effect, we again fit a GLMM with a binomial distribution and a logit link. Again, genotypes differed significantly in oviposition preferences (χ^2^ = 33.25, p = 0.0009); the model specified individual covariance parameters for each genotype, resulting in no apparent overdispersion (Generalized χ^2^/DF  = 1.00). Ethanol concentration significantly predicted female preference for the y^-^ side (F_1,562.4_ = 100.36, p<0.0001). Females were 2.5 times (95% CL: 1.7–3.8) more likely to lay on y^-^ when both sides contained 0.8% ethanol, relative to their preferences when there was no ethanol present. Genotypes also differed additively in oviposition preference (F_12,301.3_ = 14.46, p<0.0001). There was a significant genotype-by-ethanol effect (F_12,301.3_ = 3.01, p = 0.0005), indicating that the relationship between ethanol concentration and preference for y^-^ differed across genotypes (fig. 7).

Finally, ten genotypes were assayed at varying concentrations of acetic acid (0.4% and 0.0%). The relative preferences for y^-^and y^+^ media were difficult to assess, however, as flies laid very few eggs in either media with reduced acetic acid. Seven of the genotypes tested laid zero eggs in all replicates in the 0.0% condition, whereas these genotypes all oviposited in the 0.8% acetic acid condition. We therefore considered acetic acid to be indispensable, and did not attempt to quantify genotype and genotype-by-environment effects for this variable.

## Discussion

Here, we have used a simple assay to quantify the oviposition behaviors of many natural genotypes of *Drosophila melanogaster*. Surprisingly, when presented with two options, nutritious substrate (y^+^) and non-nutritious substrate (y^-^), we find that most genotypes prefer to oviposit in the less nutritious substrate. This behavior is highly polymorphic, however, with genotypes varying from 100% to nearly 10% of their eggs in y^-^ substrate. Estimates of heritability among inbred lines suggest that a considerable fraction (48.8%) of this variation is genetic, and artificial selection among outbred individuals demonstrates very high evolvability. This may indicate that variation in oviposition preferences in this generalist species is maintained by variable or frequency-dependent selection in nature, with trade-offs resulting from a balance between the risk of larvae not finding food and the risk that they will fail to develop in rotting substrate. However, other explanations are certainly possible. The simplicity of the assay conditions, which makes it possible to quantify this behavior in a large number of individuals, also means that any hypotheses regarding the significance of this variation in nature are tentative. For example, it may be that this variation is “cryptic” in the wild, and only becomes additive (selectable) in laboratory conditions [30]. In any case, determining the genes, gene networks, and neural substrates involved in decision-making in the lab would be extremely useful for understanding analogous variation in nature. If the molecular mechanisms which contribute to these behaviors could be discovered using high-throughput laboratory quantification, targeted studies of these same processes could then assess the genetic basis of oviposition variation in nature, even if the effects of individual genetic polymorphisms was variable.

The data presented here suggest that, when female *D. melanogaster* decide where to deposit their eggs, they are making a complex decision, and integrating many aspects of environmental variation. For females to prefer y^-^ media, but only when it is adjacent to y^+^ media, flies must either compare short-range sensory modalities with long-range modalities (e.g. smelling yeast but not tasting it), or compare short-range sensory indicators with spatial memory of the position of other substrates. We feel the second explanation is more likely, as some genotypes behaved very differently when options were only 1-cm apart compared to 0-cm, and again very differently at 6-cm compared to 1-cm (fig. 6). This hypothesis is perhaps supported by the apparent effect of the shape of the assay container on preference at 0-cm as well. In addition, females alter their decisions regarding yeast extract when the overall ethanol concentration changes, indicating that the concentration of ethanol alters their assessment regarding yeast. These interactions between environmental variables highlight the utility of first using a minimal media to quantify the molecular basis of decision-making behavior, and increasing ecological complexity as interactions are subsequently understood.

Recent advances in sequencing technology have created the opportunity to amass impressive amounts of data regarding genetic polymorphisms [31], [32], [33]. Understanding how this variation is maintained, and how it is utilized (or tolerated) by biological systems, depends on linking it to variation in phenotype. Our ability to mechanistically understand the morphology, behavior, and development of organisms also depends on our ability to analyze perturbations to the system, which are readily provided by natural polymorphisms. Here, we have taken the first steps towards understanding the basis of natural variation in decision-making behavior in *Drosophila* by quantifying genetic and environmental effects on these decisions.

## Methods

### Fly stocks

Inbred genotypes are maintained in non-overlapping generations in 37 ml polypropylene vials on agar-cornmeal-molasses-killed yeast medium (∼6 ml per vial). To acquire females for the oviposition assay, 5 to 8 females and males were selected and placed in a fresh food vial with a small amount of live yeast. The flies were allowed to lay eggs for 2 to 3 days at 25°C on a 12 hour light:dark cycle; the adult flies were then destroyed, and vials were kept at 25°C on a 12 hour light:dark cycle. Twelve to thirteen days post egg laying, mature offspring were anesthetized with CO_2_ and females were selected for the subsequent assays.

An outbred population was created by cross-mating virgin females and males from 173 of the inbred lines from the RAL collection. One to four males and one to four virgin females were collected from each inbred genotype (540 flies in total), and these males and females were haphazardly mixed into vials with five males and five females per vial (no males were placed in vials with females from their own genotype, insuring that all F1 offspring were mixed genotypes). For the following seven generations, all offspring were collected and mixed, and approximately 500 males and 500 females were haphazardly allocated across 100 individual vials. These vials were maintained in the same manner described above for inbred lines.

### Oviposition Assay

All female flies to be used in assays were anesthetized 24 hours before testing began and placed singly in 8 ml polypropylene test tubes containing 0.5 ml of agar-cornmeal-molasses-killed yeast medium. To test oviposition choices, female flies were then gently tapped into a 170 cc square bottom polypropylene bottle. A 35 mm petri dish lid, containing the two oviposition media, was fitted to the mouth of this bottle. The petri dish then served as both a bottle closure and an oviposition substrate container. This bottle was then placed in an inverted position (so that the petri dish lid was the base).

To prepare each petri dish lid, a steel razor blade was used as a divider: 2 ml of media were pipetted into each side of the divided lid, and blades were removed after media hardened. This left no gap between the two substrates, as the agar media expanded to occupy the space where the blade had been. Media were prepared in large batches; non-yeast media were prepared with 1% Bacto agar by weight (Difco), 0.8% ethanol by volume, and 0.8% acetic acid by volume in water; ethanol and acetic acid were added after media had cooled. Yeast-containing media was prepared the same, but contained 1% Bacto yeast extract by weight (Difco). Flies were allowed to oviposit for ∼16 hours from 5 pm to 9 am at 25°C and ∼50% humidity. The eggs laid on each type media were then counted by hand under magnification.

### Distance Oviposition Assay

Female flies to be used in the assay were collected in the same manner described above, except 4 females were placed in each test tube rather than one. The media were prepared as above and added to petri dish lids but only one media was placed on each side of the razor blade in each dish. This insured that no yeast media would contact the yeast-free media on any surface. The two media were carefully removed from the small petri dish lids and placed in the bottom half of a 150 ml square petri dish. They were separated by a distance of 0 cm (in contact, as in the other assay) to 8 cm. The square petri dish lid was placed on top and the female flies were aspirated into the dish through a small hole that was then sealed. Flies were allowed to oviposit overnight for ∼16 hours at 25°C at ∼50% humidity. The eggs laid on each type media were then counted by hand under magnification.
